# Small-cell lung cancer with a rare epidermal growth factor receptor gene mutation showing “wax-and-wane” transformation

**DOI:** 10.1186/1471-2407-13-529

**Published:** 2013-11-07

**Authors:** Yusuke Takagi, Yoshiro Nakahara, Yukio Hosomi, Tsunekazu Hishima

**Affiliations:** 1 Department of Thoracic Oncology and Respiratory Medicine, Tokyo Metropolitan Cancer and Infectious Diseases Center Komagome Hospital, 3-18-22, Honkomagome, Bunkyo-ku, Tokyo 113-8677, Japan; 2 Department of Pathology, Tokyo Metropolitan Cancer and Infectious Diseases Center Komagome Hospital, Tokyo, Japan

**Keywords:** Adenocarcinoma, Biopsy, Epidermal growth factor receptor, Erlotinib, Mutation, Pro-gastrin releasing peptide, Sialyl Lewis X antigen, Small-cell lung cancer, Transformation, Tumor marker

## Abstract

**Background:**

Small-cell lung cancer with epidermal growth factor receptor (EGFR) gene mutation typically manifests as a transformation occurring after EGFR tyrosine kinase inhibitor therapy for adenocarcinoma with *EGFR* mutation, whereas primary small-cell lung cancer showing *EGFR* mutation is extremely rare. Second biopsy of *EGFR*-mutated tumor has been broadly recognized as necessary, but is not always performed in daily practice, mainly due to the imbalance between the potential risk of the diagnostic procedure and the therapeutic impact of the biopsy result.

**Case presentation:**

A 70-year-old woman who had never smoked was referred to our hospital with chief complaints of cough and back pain. Transbronchial lung biopsy from the primary tumor of the left upper lobe revealed combined small-cell lung cancer and adenocarcinoma, a subtype of small-cell lung cancer. *EGFR* L861Q mutation was detected in both small-cell lung cancer and adenocarcinoma components. Given the staging of cT2aN3M1b (Stage IV) and histological diagnosis, first-line chemotherapy with cisplatin plus irinotecan was initiated, and partial response was achieved. Seven months after initial diagnosis, the primary tumor enlarged again, and a second biopsy from the enlarged lesion detected only adenocarcinoma with the L861Q mutation. Erlotinib was started, but multiple brain metastases and enlarged mediastinal lymph nodes subsequently appeared. Whole-brain radiation therapy was performed, and endobronchial ultrasonography-guided transbronchial biopsy from the lymph node revealed reverse transformation to small-cell lung cancer with the L861Q mutation. Amrubicin therapy achieved partial response after two cycles, with the shrinkage lasting for eight months. Serum sialyl Lewis X antigen level increased when the adenocarcinoma component was dominant, whereas plasma pro-gastrin-releasing peptide level increased when the small-cell lung cancer component became dominant.

**Conclusions:**

Transformation of the tumor correlates with the difference between small-cell lung cancer and adenocarcinoma in sensitivity to therapies, so repeated biopsies are beneficial for choosing appropriate treatments. Noninvasively obtainable parameters such as tumor markers can support the need for biopsy.

## Background

Epidermal growth factor receptor (EGFR) gene mutation is one of the most pervasive driver mutations in non-small cell lung cancer (NSCLC), particularly in adenocarcinoma [1]. Activating mutations in *EGFR* occur in exons 18 to 21, and most of the mutations in exons 18, 19 and 21 are regarded as sensitizing mutations for EGFR tyrosine kinase inhibitors (TKIs) [2]. Of these, exon 19 deletions and the exon 21 L858R point mutation account for more than 80% of mutations detected in tumors with *EGFR* mutations [2,3]. EGFR-TKI therapy for NSCLC with an *EGFR* mutation shows a significantly higher response rate, longer progression-free survival, and better quality of life when compared with platinum-doublet chemotherapy [4,5]. First-line treatment with EGFR-TKI is thus recommended for *EGFR* mutation-bearing NSCLC in recent clinical practice guidelines [6,7]. In contrast, *EGFR* mutation is rarely detected in small-cell lung cancer (SCLC). *EGFR* mutations in SCLC mostly manifest as a “transformation” after EGFR-TKI therapy in *EGFR*-mutated adenocarcinoma [8], whereas primary SCLC with *EGFR* mutation is extremely rare. Some case reports have described *EGFR*-mutated SCLC treated using EGFR-TKI, but responses to EGFR-TKI differ [9,10]. To date, whether EGFR-TKIs or cytotoxic chemotherapy should be administered for SCLC with an *EGFR* mutation remains unclear. A case of SCLC with a rare *EGFR* mutation that showed “wax-and-wane” transformation is presented, and *EGFR* mutations in SCLC are comprehensively reviewed.

## Case presentation

**Figure 1:**
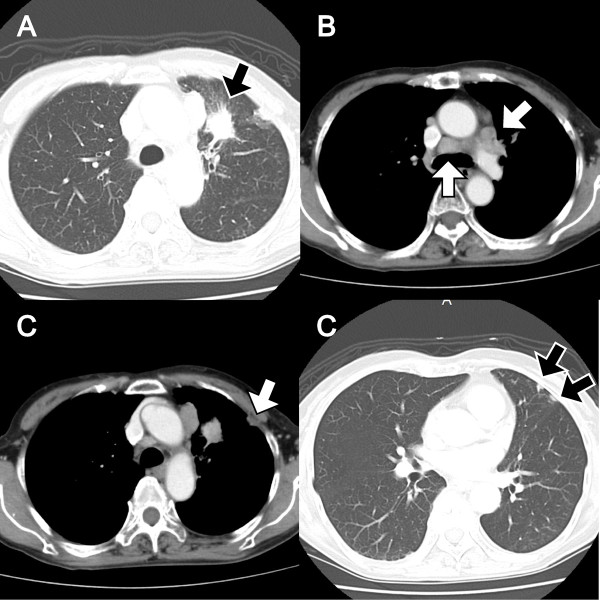
**Computed tomography at the time of initial diagnosis. A)** Primary lung cancer lesion in the upper lobe of the left lung. **B)** Enlargement of the mediastinal lymph nodes. **C)** Left pleural dissemination.

**Figure 2:**
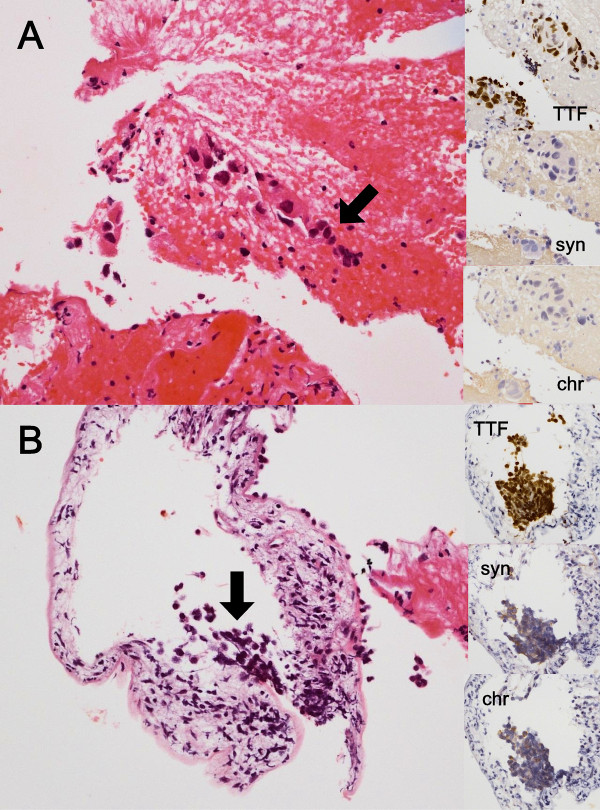
**Histopathology of combined small-cell lung cancer. A)** Transbronchial lung biopsy from the primary lesion shows adenocarcinoma (hematoxylin and eosin staining, marked with arrow). The insets show that this tumor is positive for TTF-1 and negative for synaptophysin and chromogranin. **B)** Another slice from the same biopsy specimen shows small-cell lung cancer (hematoxylin and eosin staining, marked with arrow). The insets show that this tumor is positive for TTF-1, synaptophysin, and chromogranin. chr, chromogranin; syn, synaptophysin; TTF, TTF-1.

**Figure 3:**
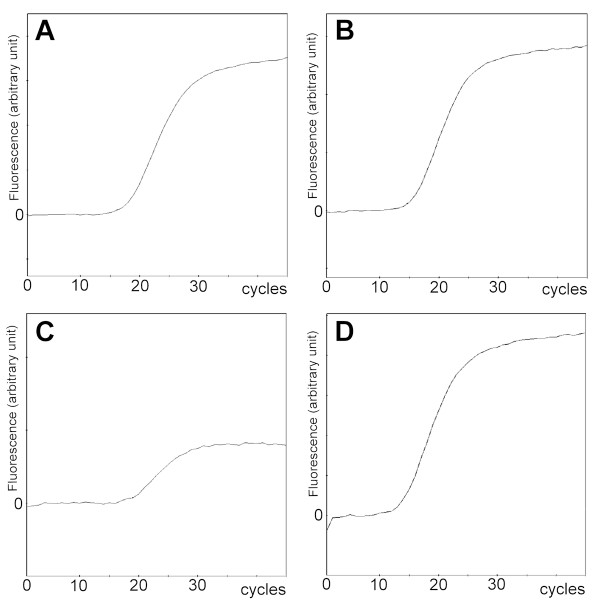
***EGFR *L861Q amplification curve for each specimen by the peptide nucleic acid-locked nucleic acid PCR clamp test. A)** Small-cell lung cancer component of the specimen at the time of initial diagnosis. **B)** Adenocarcinoma component of the specimen at the time of initial diagnosis. **C)** Adenocarcinoma at the time of disease progression after cisplatin-irinotecan therapy. **D)** Small-cell lung cancer at the time of disease progression after erlotinib therapy.

A 70-year-old Japanese woman was referred to our hospital with chief complaints of cough and back pain. She had never smoked and had no history of malignancy. Computed tomography (CT) revealed a 4-cm-diameter mass in the left upper lobe, enlargement of mediastinal lymph nodes, and left pleural dissemination (Figure 1). Asymptomatic brain metastasis was also detected on magnetic resonance imaging. A transbronchial lung biopsy (TBLB) specimen from the left upper lobe showed combined SCLC and adenocarcinoma (Figure 2), and the TNM classification of the tumor was cT2aN3M1b(BRA). The TBLB specimens were analyzed using a peptide nucleic acid-locked nucleic acid PCR clamp test, and *EGFR* exon 21 L861Q mutations were detected in both SCLC and adenocarcinoma components (Figure 3).

The patient underwent four cycles of chemotherapy comprising cisplatin (60 mg/m^2^) and irinotecan (60 mg/m^2^). The total effect of chemotherapy was partial response, and symptoms resolved. Serum sialyl Lewis X antigen (SLX) level decreased from 35 U/mL at the initiation of chemotherapy to 5.2 U/mL after four cycles of chemotherapy, and plasma levels of pro-gastrin releasing peptide (pro-GRP) also decreased from 695 pg/mL to 102 pg/mL. Complete remission of the brain metastasis was also achieved, and whole-brain radiation therapy (WBRT) was postponed at the request of the patient.

Seven months after initial diagnosis, disease progression with enlargement of the primary tumor and lymph nodes was observed. Serum SLX level was elevated to 14 U/mL, while plasma pro-GRP level remained stable (102 pg/mL). TBLB of the primary tumor was performed again, and only adenocarcinoma with the *EGFR* L861Q mutation was detected (Figure 3). Treatment with erlotinib (150 mg daily) was thus started.

After two months of disease stabilization, multiple brain metastases developed without any symptoms. The patient underwent WBRT of 30 Gy. Soon after the completion of WBRT, mediastinal lymph node enlargement occurred again. Serum SLX level was decreased (9.1 U/mL), but plasma pro-GRP level increased to 133 pg/mL. Endobronchial ultrasonography-guided transbronchial biopsy was performed. At that time, only SCLC with the *EGFR* L861Q mutation was identified (Figure 3). *PIK3CA* mutation analysis of the second (adenocarcinoma) and third (SCLC) biopsy specimens was performed, but no mutation was detected from either sample. Erlotinib was stopped, and chemotherapy with amrubicin (35 mg/m^2^) achieved partial response after two cycles. This treatment was therefore continued for nine cycles without disease progression. SLX and pro-GRP levels both decreased, to <2.5 U/mL and 79.8 pg/mL, respectively. However, chemotherapy was terminated when performance status decreased after lumbar vertebral compression without bone metastasis. Chemotherapy was then terminated. The patient remains alive as of 21 months after diagnosis.

## Conclusions

Few case reports have described primary *EGFR*-mutated adenocarcinoma transforming to SCLC and then reverse-transforming to adenocarcinoma [8,11]. As far as we can determine, this represents the first report of initially diagnosed SCLC with an *EGFR* mutation that showed transformations in both directions. *EGFR* L861Q mutation was detected from all specimens obtained on each occasion. Taking the rarity of L861Q mutation among *EGFR* activating mutations [2] into account, it appears very likely that both the SCLC and adenocarcinoma components shared a common origin. Relatively good response to cytotoxic chemotherapy was observed throughout the course of treatment. Tumor markers such as SLX and pro-GRP reflected the pathological changes in the tumor.

Although the objective response to erlotinib therapy was modest, the dominant component of the tumor in the present case changed from adenocarcinoma to SCLC after EGFR-TKI therapy. Generally, histological examination of TBLB specimens can provide the histology of only a part of the disease, but all biopsies were performed from enlarging lesions, reflecting the dominant component of the progressing disease. We suggest that responsiveness to EGFR-TKI varies between SCLC and adenocarcinoma, and the SCLC component that did not respond to erlotinib progressed selectively. In a similar manner, most cases of SCLC with *EGFR* mutations have been reported as developing acquired resistance to EGFR-TKI, and were initially diagnosed as NSCLC [8,11-15]. In general, lung adenocarcinoma and SCLC show different genetic characteristics [15,16]. In mouse models, concomitant knockout of *Rb* and *p53* causes a high incidence of SCLC, whereas mice with somatic inactivation of *p53* alone develop adenocarcinomas [17]. A previous study showed an acquired *PIK3CA* mutation in SCLC transformed from *EGFR*-mutated adenocarcinoma after EGFR-TKI therapy [8]. Unfortunately, it was not possible to detect concomitant molecular alterations in the present case, but oncogenic drivers out of *EGFR* may explain the resistance to EGFR-TKI in *EGFR*-mutated SCLC.

SCLC with an *EGFR* mutation is often resistant to EGFR-TKI, as mentioned above, whereas most cytotoxic chemotherapies achieve good response (Table 1). Second biopsy after cytotoxic chemotherapy for SCLC with an *EGFR* mutation revealed transformation to adenocarcinoma in various reports [8,11], indicating that the SCLC component is more sensitive to cytotoxic chemotherapy than the adenocarcinoma component. The differences in effectiveness of therapeutic regimens between histological types strongly support the indication of repeated biopsies at the time of each treatment change.

**Table 1:** Small-cell lung cancer with EGFR mutations and treatment outcomes

**Prior TKI**	**Mutation**	**Chemotherapy**	**PFS**	**Hist after Tx**	**Ref.**
None	L861Q	CDDP + CPT-11	7 mo	Adenocarcinoma	*
E	L861Q	AMR	8 mo	N/A	*
None	G719A	G	N/A (PR)	N/A	[9]
None	Ex19del	CRT	7 mo	N/A	[10]
None	Ex19del	CDDP + VP-16	6 mo	N/A	[10]
G	Ex19del	G	5 mo	N/A	[18]
G, E	Ex19del	G + VP-16	N/A (PD)	N/A	[12]
G	Ex19del	CDDP + CPT-11	6 mo	Adenocarcinoma	[11]
G, E	L858R	TOP	>4 mo (CR)	N/A	[13]
E	L858R	CRT	6 mo	Adenocarcinoma	[8]
E	L858R	CDDP + VP-16	N/A (PR)	N/A	[8]
G	L858R	CDDP + VP-16	N/A (PR)	N/A	[14]

Reports without sufficient information about treatment are not included on this table.

*, present case; *AMR*, Amrubicin; *CDDP*, Cisplatin; *CPT-11*, Irinotecan; *CR*, Complete response; *CRT*, Combined chemoradiotherapy; *E*, Erlotinib; *Ex19del*, Exon 19 deletions; *G*, Gefitinib; *Hist after Tx*, Histology after treatment; *mo*, Months; *N/A*, Not assessed; *PFS*, Progression-free survival; *PR*, Partial response; *Ref*., Reference; *TKI*, Epidermal growth factor receptor tyrosine kinase inhibitor; *TOP*, Topotecan; *VP-16*, Etoposide.

In this case, transitions in tumor markers (SLX and pro-GRP) appeared to occur in parallel with histological transformation. Second biopsy has been broadly recognized as necessary [8], but has not always been carried out in daily practice. This is mainly attributable to the imbalance between the potential risk of the diagnostic procedure and the therapeutic impact of the biopsy result. Given the sensitivity of EGFR-mutated SCLC to cytotoxic chemotherapies, re-biopsy for detecting tumor transformation can prove highly beneficial for patients. Noninvasive methods, including tumor markers, circulating tumor cells [19], and highly sensitive mutation detection using plasma samples [20] may play an important role in guiding the decisions of physicians and patients for the next diagnostic step, particularly when an invasive procedure is needed for obtaining tumor samples.

In conclusion, the optimal regimen for SCLC with an *EGFR* mutation cannot be uniformly defined, and should be decided according to the dominant histology at each point in the treatment course. Repeated biopsies are sometimes difficult in daily practice, but noninvasively obtainable parameters such as tumor markers can support the need for diagnostic procedures. Detailed examination of combined SCLC and adenocarcinoma, including comprehensive genome analysis, may reveal the factors that determine the histological and clinical characteristics of these tumors.

### Ethics statement

This case study was approved by the ethics committee of Tokyo Metropolitan Cancer and Infectious Diseases Center Komagome Hospital (Tokyo, Japan), and conducted in accordance with the Declaration of Helsinki. Written informed consent was obtained from the patient for publication of this Case report and any accompanying images. A copy of the written consent is available for review by the Editor of this journal.

## Competing interests

The authors declare that they have no competing interests.

## Authors’ contributions

YT was responsible for clinical management of the patient, acquisition of data, and drafting the manuscript; YN, YH and TH were responsible for interpretation of data and critical revision of the manuscript. All authors read and approved the final manuscript.

## Pre-publication history

The pre-publication history for this paper can be accessed here:

http://www.biomedcentral.com/1471-2407/13/529/prepub

## References

[B1] Kris MG Johnson BE Kwiatkowski DJ Iafrate AJ Wistuba II Aronson SL Engelman JA Shyr Y Khuri FR Rudin CM Garon EB Pao W Schiller JH Haura EB Shirai K Giaccone G Berry LD Kugler K Minna JD Bunn PA Identification of driver mutations in tumor specimens from 1,000 patients with lung adenocarcinoma: the NCI’s Lung Cancer Mutation Consortium (LCMC) J Clin Oncol 2011 29 Suppl-May CRA7506

[B2] de Pas T Toffalorio F Manzotti M Fumagalli C Spitaleri G Catania C Delmonte A Giovannini M Spaggiari L de Braud F Barberis M Activity of epidermal growth factor receptor-tyrosine kinase inhibitors in patients with non-small cell lung cancer harboring rare epidermal growth factor receptor mutations J Thorac Oncol 2011 6 1895 1901 10.1097/JTO.0b013e318227e8c6 21841502

[B3] Pao W Chmielecki J Rational, biologically based treatment of EGFR-mutant non-small-cell lung cancer Nat Rev Cancer 2010 10 760 774 10.1038/nrc2947 20966921 PMC3072803

[B4] Maemondo M Inoue A Kobayashi K Sugawara S Oizumi S Isobe H Gemma A Harada M Yoshizawa H Kinoshita I Fujita Y Okinaga S Hirano H Yoshimori K Harada T Ogura T Ando M Miyazawa H Tanaka T Saijo Y Hagiwara K Morita S Nukiwa T North-East Japan Study Group: Gefitinib or chemotherapy for non-small-cell lung cancer with mutated EGFR N Eng J Med 2010 362 2380 2388 10.1056/NEJMoa0909530 20573926

[B5] Rosell R Carcereny E Gervais R Vergnenegre A Massuti B Felip E Palmero R Garcia-Gomez R Pallares C Sanchez JM Porta R Cobo M Garrido P Longo F Moran T Insa A de Marinis F Corre R Bover I Illiano A Dansin E de Castro J Milella M Reguart N Altavilla G Jimenez U Provencio M Moreno MA Terrasa J Spanish Lung Cancer Group in collaboration with Groupe Français de Pneumo-Cancérologie and Associazione Italiana Oncologia Toracica: Erlotinib versus standard chemotherapy as first-line treatment for European patients with advanced EGFR mutation-positive non-small-cell lung cancer (EURTAC): a multicentre, open-label, randomised phase 3 trial Lancet Oncol 2012 13 239 246 10.1016/S1470-2045(11)70393-X 22285168

[B6] Peters S Adjei AA Gridelli C Reck M Kerr K Felip E ESMO Guidelines Working Group: Metastatic non-small-cell lung cancer (NSCLC): ESMO Clinical Practice Guidelines for diagnosis, treatment and follow-up Ann Oncol 2012 23 Suppl 7 vii56 vii64 10.1093/annonc/mds226 22997455

[B7] de Marinis F Rossi A di Maio M Ricciardi S Gridelli C Italian Association of Thoracic Oncology: Treatment of advanced non-small-cell lung cancer: Italian Association of Thoracic Oncology (AIOT) clinical practice guidelines Lung Cancer 2011 73 1 10 10.1016/j.lungcan.2011.02.022 21440325

[B8] Sequist LV Waltman BA Dias-Santagata D Digumarthy S Turke AB Fidias P Bergethon K Shaw AT Gettinger S Cosper AK Akhavanfard S Heist RS Temel J Christensen JG Wain JC Lynch TJ Vernovsky K Mark EJ Lanuti M Iafrate AJ Mino-Kenudson M Engelman JA Genotypic and histological evolution of lung cancers acquiring resistance to EGFR inhibitors Sci Transl Med 2011 3 75ra26 10.1126/scitranslmed.3002003 21430269 PMC3132801

[B9] Tatematsu A Shimizu J Murakami Y Horio Y Nakamura S Hida T Mitsudomi T Yatabe Y Epidermal growth factor receptor mutations in small cell lung cancer Clin Cancer Res 2008 14 6092 6096 10.1158/1078-0432.CCR-08-0332 18829487

[B10] Shiao TH Chang YL Yu CJ Chang YC Hsu YC Chang SH Shih JY Yang PC Epidermal growth factor receptor mutations in small cell lung cancer: a brief report J Thorac Oncol 2011 6 195 198 10.1097/JTO.0b013e3181f94abb 21178714

[B11] Morinaga R Okamoto I Furuta K Kawano Y Sekijima M Dote K Satou T Nishio K Fukuoka M Nakagawa K Sequential occurrence of non-small cell and small cell lung cancer with the same EGFR mutation Lung Cancer 2007 58 411 413 10.1016/j.lungcan.2007.05.014 17601631

[B12] Zakowski MF Ladanyi M Kris MG Memorial Sloan-Kettering Cancer Center Lung Cancer OncoGenome Group: EGFR mutations in small-cell lung cancers in patients who have never smoked N Eng J Med 2006 355 213 215 10.1056/NEJMc053610 16837691

[B13] Alam N Gustafson KS Ladanyi M Zakowski MF Kapoor A Truskinovsky AM Dudek AZ Small-cell carcinoma with an epidermal growth factor receptor mutation in a never-smoker with gefitinib-responsive adenocarcinoma of the lung Clin Lung Cancer 2010 11 E1 E4 10.3816/CLC.2010.n.046 20837450

[B14] Ma AT Chan WK Ma ES Cheng T Cheng PN Small cell lung cancer with an epidermal growth factor receptor mutation in primary gefitinib-resistant adenocarcinoma of the lung Acta Oncol 2012 51 557 559 10.3109/0284186X.2011.636757 22129360

[B15] Imielinski M Berger AH Hammerman PS Hernandez B Pugh TJ Hodis E Cho J Suh J Capelletti M Sivachenko A Sougnez C Auclair D Lawrence MS Stojanov P Cibulskis K Choi K de Waal L Sharifnia T Brooks A Greulich H Banerji S Zander T Seidel D Leenders F Ansén S Ludwig C Engel-Riedel W Stoelben E Wolf J Goparju C Mapping the hallmarks of lung adenocarcinoma with massively parallel sequencing Cell 2012 150 1107 1120 10.1016/j.cell.2012.08.029 22980975 PMC3557932

[B16] Peifer M Fernández-Cuesta L Sos ML George J Seidel D Kasper LH Plenker D Leenders F Sun R Zander T Menon R Koker M Dahmen I Müller C di Cerbo V Schildhaus HU Altmüller J Baessmann I Becker C de Wilde B Vandesompele J Böhm D Ansén S Gabler F Wilkening I Heynck S Heuckmann JM Lu X Carter SL Cibulskis K Integrative genome analyses identify key somatic driver mutations of small-cell lung cancer Nat Genet 2012 44 1104 1110 10.1038/ng.2396 22941188 PMC4915822

[B17] Meuwissen R Linn SC Linnoila RI Zevenhoven J Mooi WJ Berns A Induction of small cell lung cancer by somatic inactivation of both Trp53 and Rb1 in a conditional mouse model Cancer Cell 2003 4 181 189 10.1016/S1535-6108(03)00220-4 14522252

[B18] Araki J Okamoto I Suto R Ichikawa Y Sasaki J Efficacy of the tyrosine kinase inhibitor gefitinib in a patient with metastatic small cell lung cancer Lung Cancer 2005 48 141 144 10.1016/j.lungcan.2004.10.012 15777982

[B19] Maheswaran S Sequist LV Nagrath S Ulkus L Brannigan B Collura CV Inserra E Diederichs S Iafrate AJ Bell DW Digumarthy S Muzikansky A Irimia D Settleman J Tompkins RG Lynch TJ Toner M Haber DA Detection of mutations in EGFR in circulating lung-cancer cells N Eng J Med 2008 359 366 377 10.1056/NEJMoa0800668 PMC3551471 18596266

[B20] Taniguchi K Uchida J Nishino K Kumagai T Okuyama T Okami J Higashiyama M Kodama K Imamura F Kato K Quantitative detection of EGFR mutations in circulating tumor DNA derived from lung adenocarcinomas Clin Cancer Res 2011 17 7808 7815 10.1158/1078-0432.CCR-11-1712 21976538

